# Supplementary material for: Effect of Ballroom Dancing on the Physical, Psychological, and Mental Well-Being of Oncological Patients: A Pilot Study
Source: Int J Environ Res Public Health. 2025 Mar 21;22(4):470. doi: 10.3390/ijerph22040470 (PMC12026759; doi:10.3390/ijerph22040470)
Supplement: Supplementary file 1 [file ijerph-22-00470-s001.zip › ijerph-3375494-supplementary.pdf]

Figure S1. Questionnaire.

Pseudonym:

1st letter First name ____

1st letter Last name ____

Year of birth ____

1. **Personal details**

Age: o <30 years o 41-55 years o 66-75 years
o 31-40 years o 56-65 years o >75 years

Gender: o female o male

You are: o patient o healthy partner

What kind of cancer are you suffering from?

____

How long ago did you find out that you were suffering from cancer?
o < 1 month o <1 year
o > 1 year o >5 years

Are you currently receiving treatment for cancer?
o yes o no

If yes, which?
o Radiotherapy
o Medical therapy
 o Chemotherapy
 o Hormontherapy
 o Other
 o I don't know

Who are you attending with?
o partner o family
o friend o alone

Do you have any dance experience?
o no o yes
If yes, for how long? ______

Figure S2. Psychological well-being of all participants.

Comparison of mean psychological well-being of all participants (N=51)

Comparison	Mean difference	Standard Error	p-value	Lower 95% confidence bound	Upper 95% confidence bound
Before - after	1.1783	0.1093	<.0001	0.9638	1.3928
Before - evening	1.0826	0.1093	<.0001	0.8681	1.2971
Before - day1	0.7913	0.1093	<.0001	0.5768	1.0058
Before - day2	0.6870	0.1093	<.0001	0.4725	0.9015
Before - day3	0.3826	0.1093	0.0005	0.1681	0.5971
Before - day4	0.3266	0.1096	0.0030	0.1116	0.5417
Before - day5	0.2126	0.1096	0.0527	-0.00246	0.4276
Before - day6	0.2009	0.1098	0.0677	-0.01464	0.4165

Means of psychological well-being of all participants (N=51)

Time	Mean	Standard Error	p-value	Lower 95% confidence bound	Upper 95% confidence bound
before	4.4522	0.1444	<.0001	4.1689	4.7355
after	3.2739	0.1444	<.0001	2.9906	3.5572
evening	3.3696	0.1444	<.0001	3.0863	3.6529
day1	3.6609	0.1444	<.0001	3.3776	3.9442
day2	3.7652	0.1444	<.0001	3.4819	4.0485
day3	4.0696	0.1444	<.0001	3.7863	4.3529
day4	4.1256	0.1446	<.0001	3.8418	4.4093
day5	4.2396	0.1446	<.0001	3.9559	4.5233
day6	4.2512	0.1448	<.0001	3.9671	4.5354

Figure S3. Mental well-being of all participants.

Comparison of mean mental well-being of all participants (N=51)

Comparison	Mean difference	Standard Error	p-value	Lower 95% confidence bound	Upper 95% confidence bound
Before - after	0.8157	0.1024	<.0001	0.6147	1.0168
Before - evening	0.9528	0.1022	<.0001	0.7521	1.1534
Before - day1	0.7919	0.1022	<.0001	0.5912	0.9926
Before - day2	0.5788	0.1022	<.0001	0.3782	0.7795
Before - day3	0.5310	0.1022	<.0001	0.3304	0.7317
Before - day4	0.4222	0.1025	<.0001	0.2211	0.6234
Before - day5	0.3301	0.1025	0.0013	0.1290	0.5313
Before - day6	0.3257	0.1025	0.0015	0.1246	0.5269

Means of mental well-being of all participants (N=51)

Time	Mean	Standard Error	p-value	Lower 95% confidence bound	Upper 95% confidence bound
before	4.6875	0.1490	<.0001	4.3952	4.9799
after	3.8718	0.1488	<.0001	3.5798	4.1638
evening	3.7348	0.1486	<.0001	3.4431	4.0265
day1	3.8957	0.1486	<.0001	3.6039	4.1874
day2	4.1087	0.1486	<.0001	3.8170	4.4004
day3	4.1565	0.1486	<.0001	3.8648	4.4482
day4	4.2653	0.1488	<.0001	3.9733	4.5574
day5	4.3574	0.1488	<.0001	4.0654	4.6495
day6	4.3618	0.1488	<.0001	4.0697	4.6539

Figure S4. Comparison of mean physical well-being by time and gender.

Comparison of mean physical well-being by time and gender (N=43, Female=34, Male=9)

Comparison	Mean difference	Standard Error	p-value	Lower 95% confidence bound	Upper 95% confidence bound
Before: Female - Male	0.6804	0.3571	0.0571	-0.02056	1.3813
After: Female - Male	0.8376	0.3571	0.0192	0.1367	1.5385
Evening: Female - Male	0.6344	0.3601	0.0785	-0.07245	1.3413
Day1: Female - Male	0.9191	0.3571	0.0102	0.2182	1.6200
Day2: Female - Male	0.7330	0.3571	0.0404	0.03207	1.4339
Day3: Female - Male	0.7526	0.3571	0.0354	0.05165	1.4535
Day4: Female - Male	0.4787	0.3574	0.1808	-0.2228	1.1803
Day5: Female - Male	0.6283	0.3572	0.0790	-0.07290	1.3296
Day6: Female - Male	0.3964	0.3572	0.2675	-0.3049	1.0976
Female: Before - after	1.1585	0.1274	<.0001	0.9085	1.4085
Before - evening	1.2744	0.1274	<.0001	1.0244	1.5244
Before - day1	1.0244	0.1274	<.0001	0.7744	1.2744
Before - day2	1.0000	0.1274	<.0001	0.7500	1.2500
Before - day3	0.6646	0.1274	<.0001	0.4146	0.9146
Before - day4	0.6227	0.1283	<.0001	0.3709	0.8745
Before - day5	0.4204	0.1278	0.0010	0.1696	0.6713
Before - day6	0.4945	0.1278	0.0001	0.2436	0.7454
Male: Before - after	1.3158	0.2646	<.0001	0.7964	1.8352
Before - evening	1.2285	0.2687	<.0001	0.7011	1.7558
Before - day1	1.2632	0.2646	<.0001	0.7438	1.7825
Before - day2	1.0526	0.2646	<.0001	0.5333	1.5720
Before - day3	0.7368	0.2646	0.0055	0.2175	1.2562
Before - day4	0.4211	0.2646	0.1119	-0.09832	0.9404
Before - day5	0.3684	0.2646	0.1642	-0.1509	0.8878
Before - day6	0.2105	0.2646	0.4265	-0.3088	0.7299

Figure S5. Comparison of mean psychological well-being by time and gender.

Comparison of mean psychological well-being by time and gender (N=43, Female=34, Male=9)

Comparison	Mean difference	Standard Error	p-value	Lower 95% confidence bound	Upper 95% confidence bound
Before: Female - Male	1.0879	0.3953	0.0061	0.3120	1.8638
After: Female - Male	0.8158	0.3953	0.0394	0.03990	1.5917
Evening: Female - Male	0.7776	0.3953	0.0495	0.001713	1.5535
Day1: Female - Male	0.8376	0.3953	0.0344	0.06173	1.6135
Day2: Female - Male	0.7246	0.3953	0.0671	-0.05124	1.5005
Day3: Female - Male	0.6245	0.3953	0.1145	-0.1514	1.4004
Day4: Female - Male	0.5886	0.3954	0.1370	-0.1875	1.3648
Day5: Female - Male	0.8018	0.3954	0.0429	0.02558	1.5780
Day6: Female - Male	0.3122	0.3956	0.4302	-0.4643	1.0887
Female: Before - after	1.2195	0.1326	<.0001	0.9592	1.4798
Before - evening	1.1524	0.1326	<.0001	0.8922	1.4127
Before - day1	0.8293	0.1326	<.0001	0.5690	1.0895
Before - day2	0.7317	0.1326	<.0001	0.4714	0.9920
Before - day3	0.4634	0.1326	0.0005	0.2031	0.7237
Before - day4	0.4467	0.1331	0.0008	0.1855	0.7078
Before - day5	0.2862	0.1331	0.0318	0.02497	0.5474
Before - day6	0.4073	0.1335	0.0024	0.1452	0.6694
Male: Before - after	0.9474	0.2754	0.0006	0.4067	1.4881
Before - evening	0.8421	0.2754	0.0023	0.3014	1.3828
Before - day1	0.5789	0.2754	0.0359	0.03826	1.1196
Before - day2	0.3684	0.2754	0.1814	-0.1723	0.9091
Before - day3	4.44E-16	0.2754	1.0000	-0.5407	0.5407
Before - day4	-0.05263	0.2754	0.8485	-0.5933	0.4881
Before - day5	1.33E-15	0.2754	1.0000	-0.5407	0.5407
Before - day6	-0.3684	0.2754	0.1814	-0.9091	0.1723

Figure S6. Comparison of mean mental well-being by time and gender.

Comparison of mean mental well-being by time and gender (N=43, Female=34, Male=9)

Comparison	Mean difference	Standard Error	p-value	Lower 95% confidence bound	Upper 95% confidence bound
Before: Female - Male	0.6938	0.4122	0.0927	-0.1152	1.5029
After: Female - Male	0.5632	0.4122	0.1722	-0.2458	1.3723
Evening: Female - Male	0.6454	0.4120	0.1177	-0.1634	1.4542
Day1: Female - Male	0.5995	0.4120	0.1461	-0.2093	1.4083
Day2: Female - Male	0.6268	0.4120	0.1286	-0.1821	1.4356
Day3: Female - Male	0.4506	0.4120	0.2745	-0.3582	1.2594
Day4: Female - Male	0.3186	0.4122	0.4398	-0.4905	1.1276
Day5: Female - Male	0.4761	0.4122	0.2484	-0.3329	1.2852
Day6: Female - Male	0.3153	0.4122	0.4445	-0.4938	1.1244
Female: Before - after	0.8148	0.1227	<.0001	0.5740	1.0557
Before - evening	0.9958	0.1224	<.0001	0.7556	1.2360
Before - day1	0.8312	0.1224	<.0001	0.5910	1.0714
Before - day2	0.5934	0.1224	<.0001	0.3532	0.8336
Before - day3	0.6117	0.1224	<.0001	0.3715	0.8519
Before - day4	0.5332	0.1228	<.0001	0.2921	0.7742
Before - day5	0.4282	0.1228	0.0005	0.1872	0.6693
Before - day6	0.4838	0.1228	<.0001	0.2427	0.7248
Male: Before - after	0.6842	0.2533	0.0071	0.1869	1.1815
Before - evening	0.9474	0.2533	0.0002	0.4501	1.4447
Before - day1	0.7368	0.2533	0.0037	0.2396	1.2341
Before - day2	0.5263	0.2533	0.0381	0.02903	1.0236
Before - day3	0.3684	0.2533	0.1463	-0.1289	0.8657
Before - day4	0.1579	0.2533	0.5333	-0.3394	0.6552
Before - day5	0.2105	0.2533	0.4062	-0.2868	0.7078
Before - day6	0.1053	0.2533	0.6779	-0.3920	0.6025

Figure S7. Table of Patient/Healthy by Gender.

Table of Patient/Healthy by Gender (N=45)

Table of Patient/Healthy by Gender

PvsH(PvsH) *Frequency* *Percent* *Row Pct* *Col Pct*	*Gender(FvsM)* *F*	*M*	*Total*
Patient	34	3	37
	75.56	6.67	82.22
	91.89	8.11	
	91.89	37.50	
Healthy	3	5	8
	6.67	11.11	17.78
	37.50	62.50	
	8.11	62.50	
Total	37	8	45
	82.22	17.78	100.00

Frequency Missing = 7

Figure S8. Comparison of mean physical well-being by time and patient/healthy.

Comparison of mean physical well-being by time and patient/healthy (N=50, patient=38, healthy=12)

Comparison	Mean difference	Standard Error	p-value	Lower 95% confidence bound	Upper 95% confidence bound
Before: Patient - Healthy	0.7610	0.3248	0.0193	0.1236	1.3984
After: Patient - Healthy	0.5577	0.3248	0.0863	-0.07968	1.1951
Evening: Patient - Relative	0.2584	0.3268	0.4293	-0.3829	0.8998
Day1: Patient - Healthy	0.2761	0.3248	0.3955	-0.3613	0.9135
Day2: Patient - Healthy	0.3201	0.3248	0.3246	-0.3173	0.9574
Day3: Patient - Healthy	0.5522	0.3248	0.0894	-0.08517	1.1896
Day4: Patient - Healthy	0.3847	0.3250	0.2369	-0.2532	1.0226
Day5: Patient - Healthy	0.5469	0.3249	0.0926	-0.09071	1.1846
Day6: Patient - Healthy	0.09416	0.3249	0.7720	-0.5435	0.7318
Patient: Before - after	1.2033	0.1201	<.0001	0.9675	1.4391
Before - evening	1.3022	0.1201	<.0001	1.0664	1.5380
Before - day1	1.1099	0.1201	<.0001	0.8741	1.3457
Before - day2	1.0659	0.1201	<.0001	0.8302	1.3017
Before - day3	0.7088	0.1201	<.0001	0.4730	0.9446
Before - day4	0.6263	0.1209	<.0001	0.3890	0.8636
Before - day5	0.4224	0.1205	0.0005	0.1858	0.6589
Before - day6	0.5002	0.1205	<.0001	0.2636	0.7367
Healthy: Before - after	1.0000	0.2339	<.0001	0.5409	1.4591
Before - evening	0.7996	0.2368	0.0008	0.3350	1.2643
Before - day1	0.6250	0.2339	0.0077	0.1659	1.0841
Before - day2	0.6250	0.2339	0.0077	0.1659	1.0841
Before - day3	0.5000	0.2339	0.0328	0.04089	0.9591
Before - day4	0.2500	0.2339	0.2855	-0.2091	0.7091
Before - day5	0.2083	0.2339	0.3734	-0.2508	0.6674
Before - day6	-0.1667	0.2339	0.4764	-0.6258	0.2924

Figure S9. Comparison of mean psychological well-being by time and patient/healthy.

Comparison of mean psychological well-being by time and patient/healthy (N=50, patient=38, healthy=12)

Comparison	Mean difference	Standard Error	p-value	Lower 95% confidence bound	Upper 95% confidence bound
Before: Patient - Healthy	1.4139	0.3487	<.0001	0.7296	2.0982
After: Patient - Healthy	0.8727	0.3487	0.0125	0.1884	1.5570
Evening: Patient - Healthy	0.5723	0.3487	0.1010	-0.1119	1.2566
Day1: Patient - Healthy	0.7825	0.3487	0.0251	0.09822	1.4668
Day2: Patient - Healthy	0.8091	0.3487	0.0205	0.1248	1.4934
Day3: Patient - Healthy	0.7198	0.3487	0.0393	0.03549	1.4041
Day4: Patient - Healthy	0.6321	0.3488	0.0703	-0.05242	1.3167
Day5: Patient - Healthy	0.6710	0.3488	0.0547	-0.01353	1.3556
Day6: Patient - Healthy	0.2623	0.3489	0.4523	-0.4225	0.9472
Patient: Before - after	1.2912	0.1220	<.0001	1.0518	1.5306
Before - evening	1.2582	0.1220	<.0001	1.0188	1.4977
Before - day1	0.9231	0.1220	<.0001	0.6836	1.1625
Before - day2	0.8132	0.1220	<.0001	0.5738	1.0526
Before - day3	0.5275	0.1220	<.0001	0.2880	0.7669
Before - day4	0.4901	0.1224	<.0001	0.2499	0.7303
Before - day5	0.3679	0.1224	0.0027	0.1277	0.6081
Before - day6	0.4432	0.1228	0.0003	0.2023	0.6842
Healthy: Before - after	0.7500	0.2376	0.0016	0.2838	1.2162
Before - evening	0.4167	0.2376	0.0798	-0.04955	0.8829
Before - day1	0.2917	0.2376	0.2198	-0.1746	0.7579
Before - day2	0.2083	0.2376	0.3807	-0.2579	0.6746
Before - day3	-0.1667	0.2376	0.4831	-0.6329	0.2996
Before - day4	-0.2917	0.2376	0.2198	-0.7579	0.1746
Before - day5	-0.3750	0.2376	0.1148	-0.8412	0.09122
Before - day6	-0.7083	0.2376	0.0029	-1.1746	-0.2421

Figure S10. Comparison of mean mental well-being by time and patient/healthy.

Comparison of mean mental well-being by time and patient/healthy (N=50, patient=38, healthy=12)

Comparison	Mean difference	Standard Error	p-value	Lower 95% confidence bound	Upper 95% confidence bound
Before: Patient - Healthy	1.2932	0.3600	0.0003	0.5867	1.9996
After: Patient - Healthy	0.9973	0.3598	0.0057	0.2911	1.7035
Evening: Patient - Healthy	0.8233	0.3597	0.0223	0.1173	1.5293
Day1: Patient - Healthy	0.7633	0.3597	0.0341	0.05728	1.4693
Day2: Patient - Healthy	0.6639	0.3597	0.0653	-0.04207	1.3699
Day3: Patient - Healthy	0.6717	0.3597	0.0622	-0.03429	1.3777
Day4: Patient - Healthy	0.5980	0.3598	0.0969	-0.1082	1.3042
Day5: Patient - Healthy	0.6091	0.3598	0.0908	-0.09709	1.3154
Day6: Patient - Healthy	0.3508	0.3598	0.3299	-0.3554	1.0570
Patient: Before - after	0.8792	0.1147	<.0001	0.6541	1.1043
Before - evening	1.0533	0.1144	<.0001	0.8287	1.2778
Before - day1	0.9049	0.1144	<.0001	0.6803	1.1295
Before - day2	0.7126	0.1144	<.0001	0.4880	0.9372
Before - day3	0.6631	0.1144	<.0001	0.4386	0.8877
Before - day4	0.5702	0.1148	<.0001	0.3449	0.7955
Before - day5	0.4757	0.1148	<.0001	0.2504	0.7010
Before - day6	0.5257	0.1148	<.0001	0.3004	0.7510
Healthy: Before - after	0.5833	0.2214	0.0086	0.1488	1.0179
Before - evening	0.5833	0.2214	0.0086	0.1488	1.0179
Before - day1	0.3750	0.2214	0.0907	-0.05954	0.8095
Before - day2	0.08333	0.2214	0.7067	-0.3512	0.5179
Before - day3	0.04167	0.2214	0.8508	-0.3929	0.4762
Before - day4	-0.1250	0.2214	0.5725	-0.5595	0.3095
Before - day5	-0.2083	0.2214	0.3470	-0.6429	0.2262
Before - day6	-0.4167	0.2214	0.0602	-0.8512	0.01787
